# Exendin-4 attenuates blast traumatic brain injury induced cognitive impairments, losses of synaptophysin and *in vitro* TBI-induced hippocampal cellular degeneration

**DOI:** 10.1038/s41598-017-03792-9

**Published:** 2017-06-16

**Authors:** Lital Rachmany, David Tweedie, Vardit Rubovitch, Yazhou Li, Harold W. Holloway, Dong Seok Kim, Whitney A. Ratliff, Jessica N. Saykally, Bruce A. Citron, Barry J. Hoffer, Nigel H. Greig, Chaim G. Pick

**Affiliations:** 1 Department of Anatomy and Anthropology, Sackler School of Medicine, Tel-Aviv University, Tel-Aviv, 69978 Israel; 2 Sagol School of Neuroscience, Tel-Aviv University, Tel-Aviv, 69978 Israel; 3 The Dr. Miriam and Sheldon G. Adelson Chair and Center for the Biology of Addictive Diseases, Tel-Aviv University, Tel-Aviv, 69978 Israel; 4 Drug Design & Development Section, Translational Gerontology Branch, Intramural Research Program, National Institute on Aging, National Institutes of Health, Baltimore, MD 21224 USA; 5 Peptron Inc., 37-24, Yuseong-daero 1628 beon-gil, Yuseong-gu, Daejeon 305-811 Republic of Korea; 6 Laboratory of Molecular Biology, Research and Development 151, Bay Pines VA Healthcare System, Bay Pines, FL 33744 USA; 7 Department of Molecular Medicine, USF Morsani College of Medicine, Tampa, FL 33612 USA; 8 Department of Neurosurgery, Case Western Reserve University School of Medicine, Cleveland, OH 44106 USA

## Abstract

Mild blast traumatic brain injury (B-TBI) induced lasting cognitive impairments in novel object recognition and less severe deficits in Y-maze behaviors. B-TBI significantly reduced the levels of synaptophysin (SYP) protein staining in cortical (CTX) and hippocampal (HIPP) tissues. Treatment with exendin-4 (Ex-4) delivered by subcutaneous micro-osmotic pumps 48 hours prior to or 2 hours immediately after B-TBI prevented the induction of both cognitive deficits and B-TBI induced changes in SYP staining. The effects of a series of biaxial stretch injuries (BSI) on a neuronal derived cell line, HT22 cells, were assessed in an *in vitro* model of TBI. Biaxial stretch damage induced shrunken neurites and cell death. Treatment of HT22 cultures with Ex-4 (25 to 100 nM), prior to injury, attenuated the cytotoxic effects of BSI and preserved neurite length similar to sham treated cells. These data imply that treatment with Ex-4 may represent a viable option for the management of secondary events triggered by blast-induced, mild traumatic brain injury that is commonly observed in militarized zones.

## Introduction

Traumatic brain injury (TBI) is a common ailment that presently lacks a first line pharmacological treatment approved by the US Food and Drug Administration (U.S. FDA). TBI can be caused by various forms of injury and tends to be more common in males, children under 14, and adults over 65 years of age^1^. The most common form of TBI occurring in the civilian population is concussive in nature and is exemplified by automobile accidents and full contact sports^2^. In the setting of a military arena causes of TBI are typically more variable and may include complex combinations of concussive and/or explosive blast shockwave-induced injury that tend to be predominantly mild in nature^3, 4^. The pathology of TBI can be thought of as a set of time-dependent processes: (1) a primary event derived from damage to the brain tissues by the initiating TBI, and (2) a series of secondary events triggered in response to the primary damage, which may include neuronal excitotoxicity, the induction of neuroinflammation and apoptotic neuronal cell death^5–8^.

Numerous models of TBI have been developed into which key aspects of human TBI pathology have been incorporated. While these studies have provided insights of likely clinical pathological processes involved in human TBI, it is important to acknowledge that no one animal model truly represents human TBI^9, 10^. Due to the presence of civilians as well as military personnel in battlefield zones across the world and the increased use of ‘improvised explosive devices’ by enemy combatants^11–13^, we have, in the present study, focused our research efforts upon investigating a possible drug treatment for mild blast TBI (B-TBI).

Here we investigate a clinically translatable dose of a neurotrophic and neuroprotective U.S. FDA approved drug used for the treatment of type II diabetes mellitus, exendin-4 (Ex-4)- also known as exenatide, in the setting of a mouse model of mild B-TBI^14–16^. Ex-4 is an agonist for glucagon-like peptide 1 receptors (GLP-1R) which mediates neurotrophic and neuroprotective actions through signaling pathways downstream of the GLP-1R^17^. GLP-1Rs are widely distributed in brain and Ex-4 gains ready access to brain tissues after peripheral administration^18^.

We assessed the effects of B-TBI in the absence and presence of pre- or post-injury treatment with Ex-4 on mouse cognition at days 7 and 30 after injury. Additionally, we examined the effects of the blast on synaptophysin (SYP) staining levels, a marker for pre-synaptic neurons in cortical and hippocampal tissue 3 days after injury. Where behaviors and protein staining were altered by B-TBI we observed that treatment with Ex-4, irrespective of the timing of treatment, attenuated the effects of the blast. The effects of Ex-4 were also assessed on an *in vitro* model of TBI employing a hippocampal cell derived cell line (HT22 cells^19, 20^). The effects of TBI on cell viability and neurite length were assessed in the absence or presence of pre-treatment with Ex-4. We found that a biaxial *in vitro* cell injury can induce abnormal neurite length and cell death that is amenable to attenuation by treatment with Ex-4. Taken together, these data suggest that treatment with the U.S. FDA approved drug Ex-4 may possess clinically relevant benefits to patients who have experienced a mild blast traumatic brain injury, although further studies are required to more fully explore the potential of Ex-4 as a treatment for human blast TBI.

## Results

### Pharmacokinetic evaluation of the time dependent plasma levels of Ex-4 after pump implantation

**Figure 1:**
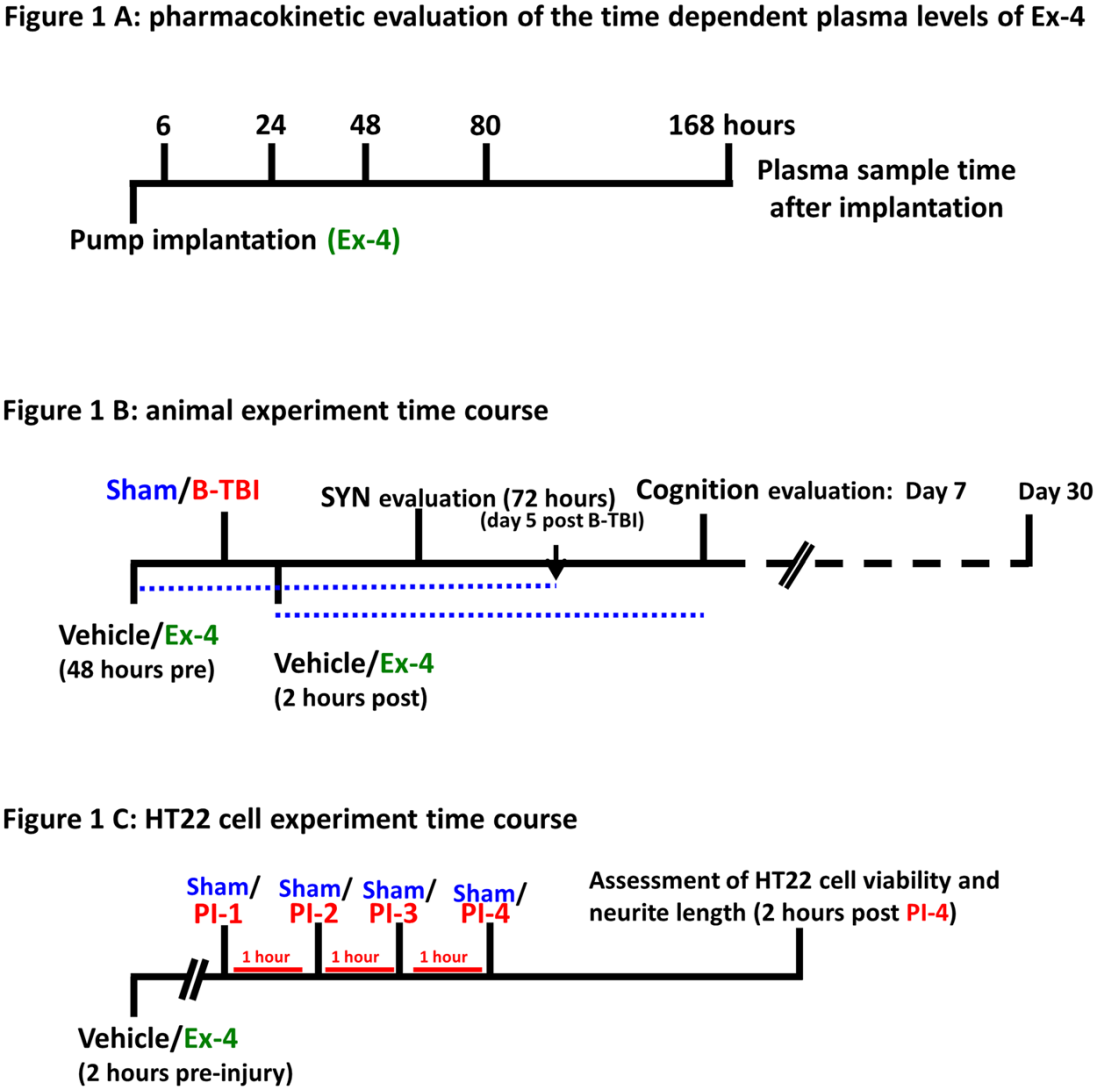
Schema of animal and cell culture study design. Panel A: Plasma was prepared from animals at the indicated times after the surgical implantation of the micro-osmotic pumps containing Ex-4. The levels of plasma Ex-4 were detected using a sensitive ELISA specific for Ex-4. Panel B: Animals were implanted with micro-osmotic pumps containing Ex-4 either 48 hours before or 2 hours after induction of a mild blast TBI (B-TBI). Brain synaptophysin staining studies were undertaken 72 hours after B-TBI. Animal cognition studies were undertaken 7 and 30 days after B-TBI. The blue dotted line indicates the duration of Ex-4 infusion by the 7 day micro-osmotic pumps. Panel C: HT22 cells were subjected to a series of sham or pulse injury (PI) procedures. Cells were pre-treated with drug vehicle or Ex-4 (25 nM or 100 nM) 2 hours prior to sham or PI challenge. Two hours after the last sham or PI challenge assessments of cell viability and neurite length were undertaken.

**Figure 2:**
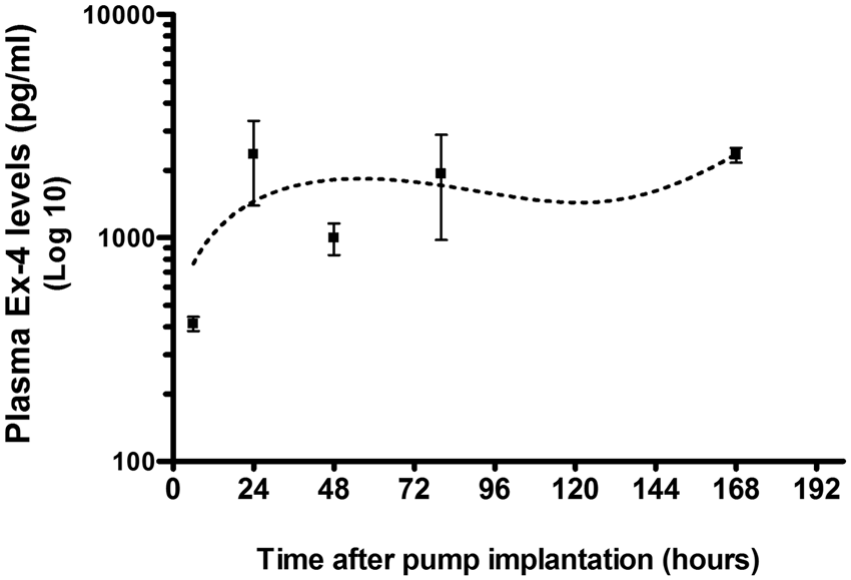
Pharmacokinetic evaluation of mouse plasma Ex-4 levels after the implantation of micro-osmotic pumps. The time dependent plasma concentrations of Ex-4 indicate a biphasic response, initially peaking at 40 hours and then at 80 hours and 7 days after micro-osmotic pump implantation. The plasma concentration (pg/ml) is indicated on the y axis (Log 10) and the sample time points after pump implantation are shown on the × axis (hours). The values are mean ± s.e.m. of n observations where n = the numbers of mice; in the present study n = 4.

Blood sample time points are illustrated in Fig. 1A. Six hours after micro-osmotic pump implantation Ex-4 was detectable in mouse plasma (414 ± 31 pg/ml). The plasma concentrations of Ex-4 were observed to increase as indicated at the additional 4 measurement time points (Fig. 2). There was an early biphasic response in the detection of plasma Ex-4 between 24 and 80 hours. Notably, the plasma Ex-4 concentrations measured at 80 hours and 7 days post-implantation were similar (1932 ± 954 pg/ml for 80 hours and 2346 ± 182 pg/ml for 7 days). Figure 1 and 2 *near here*.

### B-TBI induced changes in cognition on day 7 and 30 after injury

**Figure 3:**
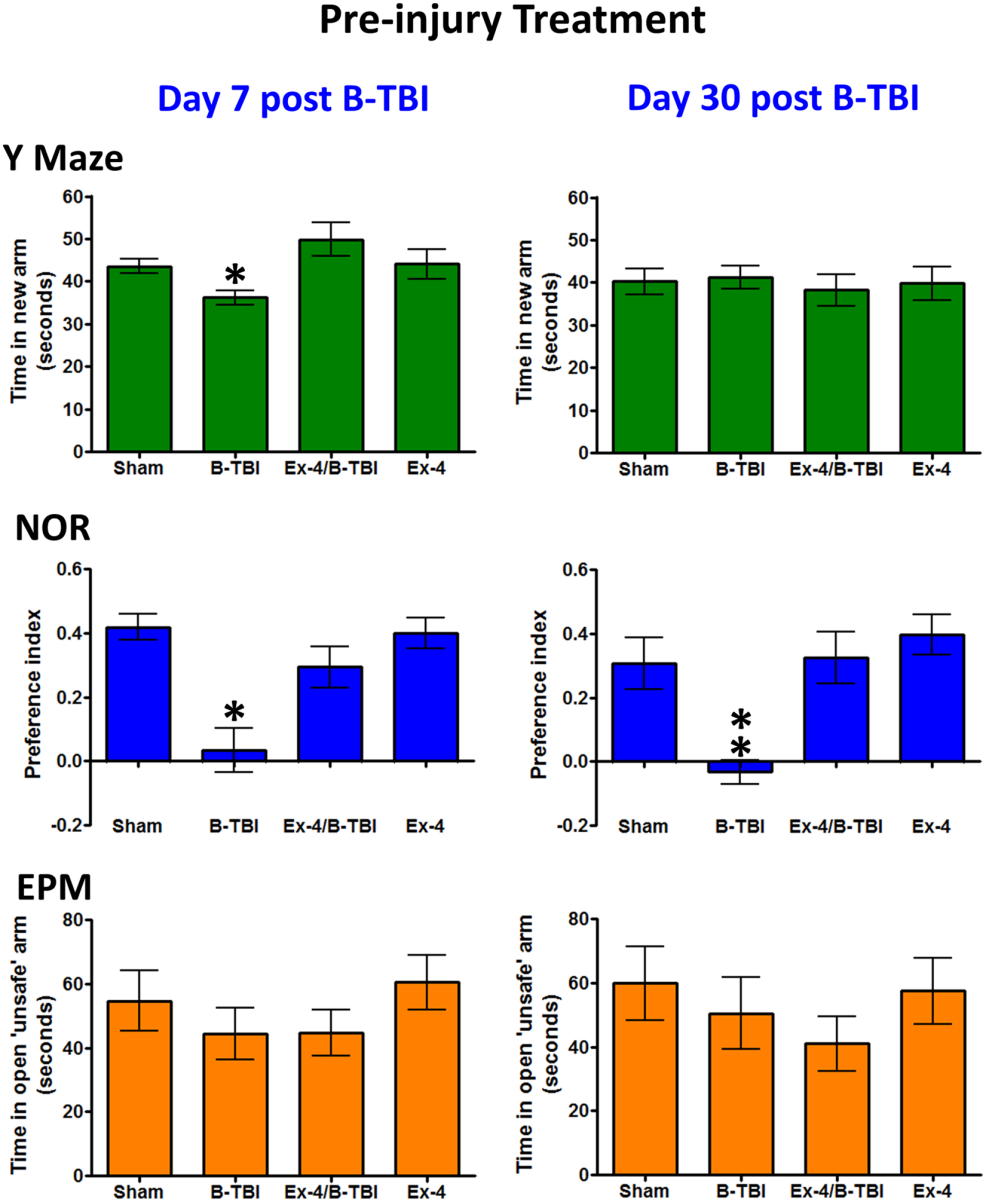
Effects of B-TBI on mouse cognition were attenuated by pre-injury treatment with Ex-4. Upper Panel: B-TBI induced a significant deficit in spatial working memory of mice at 7 days post-B-TBI (*p < 0.05); treatment with Ex-4 prevented the B-TBI-induced change in the Y Maze. Middle Panel: B-TBI animals exhibited significant impairments in object recognition memory at day 7 and 30 after B-TBI (*^,^**p < 0.05, p < 0.01). The impairments in NOR were prevented by Ex-4 at both time points. Lower Panel: Animals performed equally in EPM assessment. Data are presented as a mean ± s.e.m. of measurements. The numbers of animals per treatment group are provided in the methods section.

**Figure 4:**
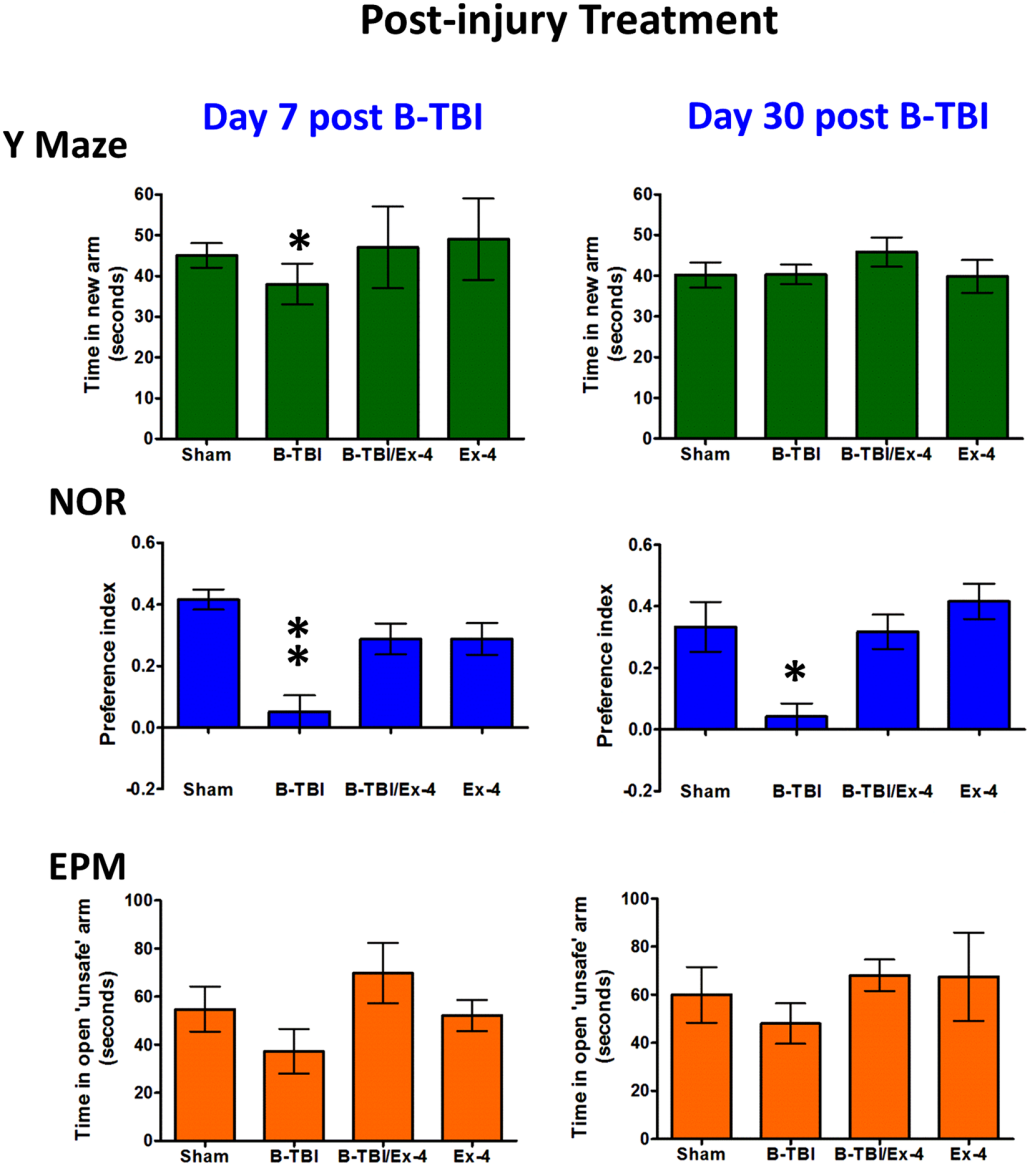
Effects of B-TBI on mouse cognition were attenuated by post-injury treatment with Ex-4. Upper Panel: B-TBI induced a significant deficit in spatial working memory of mice at 7 days post-B-TBI (*p < 0.05); treatment with Ex-4 prevented the B-TBI-induced change in the Y Maze. Middle Panel: B-TBI animals exhibited significant impairments in object recognition memory at day 7 and 30 after B-TBI (*^,^**p < 0.05, p < 0.01). The impairments in NOR were prevented by Ex-4 at both time points. Lower Panel: Animals performed equally in EPM assessment. Data are presented as a mean ± s.e.m. of measurements. The numbers of animals per treatment group are provided in the methods section.

The animal treatment time line is illustrated in Fig. 1B. Y-maze (YM), novel object recognition (NOR) and elevated plus maze (EPM) assessments were undertaken in B-TBI and Ex-4 pre- or post-injury treated animals. Evaluations were initiated on day 7, at a time point where plasma Ex-4 levels will have been at steady-state levels for both treatment regiments (Fig. 2). A separate animal cohort was evaluated at 30 days after injury; these animals only received Ex-4 during the initial 7 days of the experiment, and thus by the 30 day time point they had undergone a 23–25 day washout period for Ex-4. Under these circumstances, Ex-4 mediated behavioral improvements determined at 30 days reflect structural effects of the agent facilitated via neurotrophic/neuroprotective/ anti-inflammatory and other actions^17^ within the brain, rather than symptomatic actions, as they occurred in the absence of Ex-4 at the time of evaluation. The YM assessment, undertaken on day 7, indicated that B-TBI animals were cognitively compromised as a significant difference was observed in B-TBI animals compared to sham exposed mice (*p < 0.05). Pre-treatment of B-TBI animals with Ex-4 abolished the injury induced impairment in spatial working memory (Fig. 3 Upper Left Panel). By 30 days after injury all animals displayed the same levels of spatial memory (Fig. 3 Upper Right Panel). In contrast to the YM, B-TBI animals presented significant memory deficits in NOR measured at both day 7 (*p < 0.05) and day 30 (**p < 0.01) after injury. At both time points pre-treatment with Ex-4 prevented the B-TBI induced impairments in object recognition memory (Fig. 3 Middle Panels). The findings from the EPM indicate that B-TBI animals spent the same amount of time in the open ‘unsafe’ arms of the maze as the control animals. This was the case for B-TBI animals evaluated at both time points after injury (Fig. 3 Lower Panels). In parallel studies the animals received micro-osmotic pump implantation two hours after the induction of the blast injury. The observations of animal behaviors in this treatment regimen provided data virtually indistinguishable to that of the animals that received drug as a pre-injury treatment (Fig. 4). Figures 3 and 4 *cognition, pre- post-injury treatment near here*.

### B-TBI-induced reductions in synaptophysin immunoreactivity day 3 after injury

**Figure 5:**
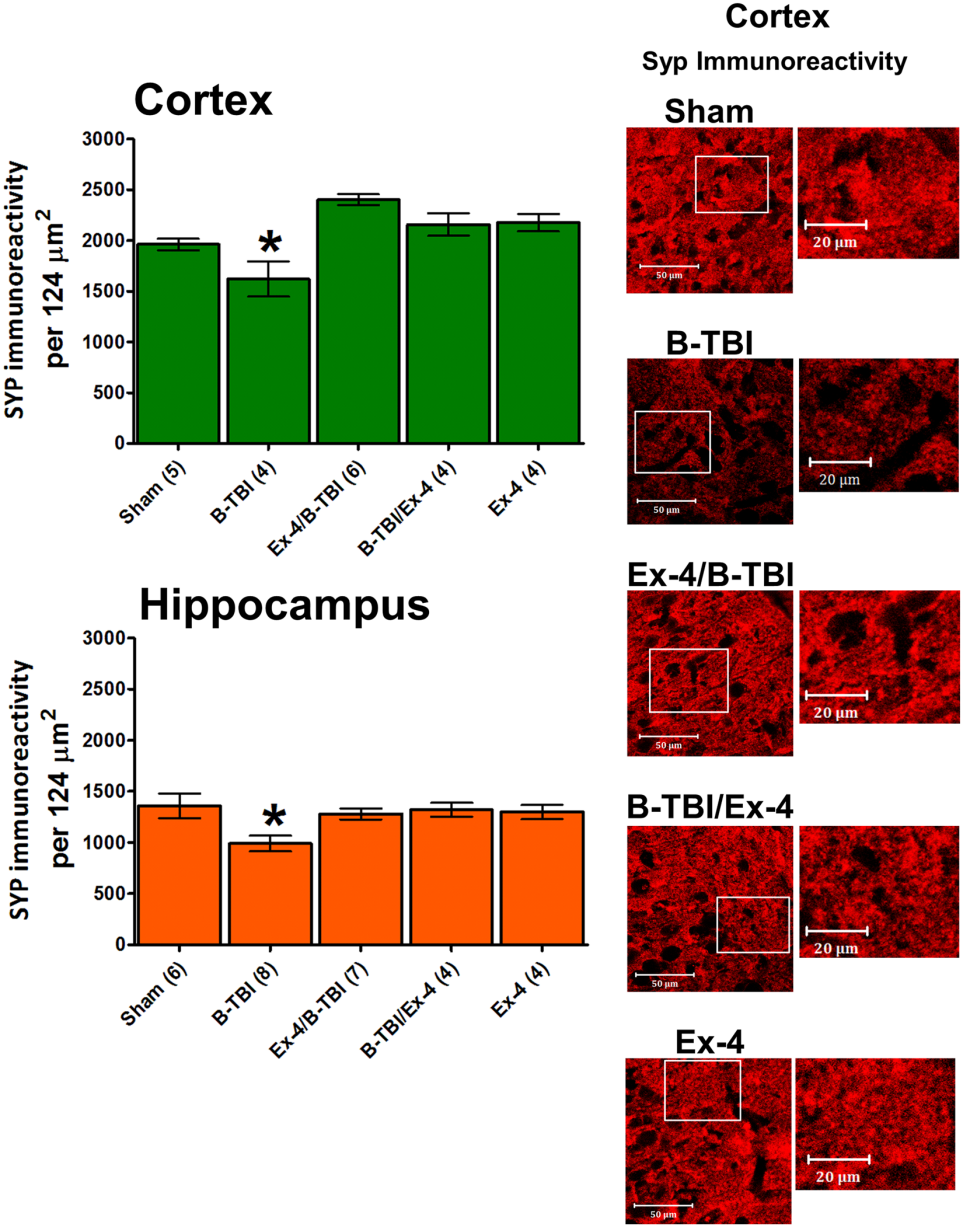
Effects of B-TBI on synaptophysin (SYP) immunoreactivity in mouse temporal cortical and hippocampal regions. Upper Left: Temporal cortex SYP immunoreactivity was significantly reduced by B-TBI (*P < 0.05). Treatment of animals with Ex-4, irrespective of time of administration, led to a preservation of immunoreactivity in B-TBI animals. Ex-4 treatment in the absence of B-TBI had no effect on SYP staining levels. Lower Left: Hippocampal SYP immunoreactivity was significantly reduced by B-TBI (*P < 0.05). Treatment of animals with Ex-4, irrespective of time of administration, led to a preservation of SYP staining in B-TBI animals. Ex-4 treatment in the absence of B-TBI had no effect on the levels of SYP immunoreactivity. Right: Representative images of SYP immunoreactivity are provided for CTX. In the left image box the scale bar = 50 µm, in the expanded higher magnification image box (right) the scale bar = 20 µm. The location of the higher magnification image is indicated in the left image box by the white box. Data are presented as a mean ± s.e.m. of measurements from n observation (numbers of animals) per treatment group; animal numbers are indicated on the x-axis labels and in the methods section.

On day 3 after B-TBI, synaptophysin (SYP) immunoreactivity was observed to be significantly lower in the temporal CTX brain region of B-TBI animals when compared to sham exposed animals (Fig. 5, Upper Left). B-TBI reduced SYP staining levels from sham control levels of 1960 ± 55 to 1574 ± 216 counts per 124 μm^2^ in cortex (a 19.7% decline, P < 0.05). Ex-4 as either a pre- or post-injury treatment can be predicted to be at a steady-state level in plasma by 72 hours after injury (Fig. 2). Ex-4 treatment was observed to prevent the reductions of SYP immunoreactivity in injured animal temporal CTX; pre-injury treatment numbers were 2216 ± 144 and post-injury treatment numbers were 2157 ± 109, vs. sham 1960 ± 55. Similar to that observed in cortex, the hippocampal area showed a significant reduction in SYP immunoreactivity when compared to control levels in B-TBI animals; SYP immunoreactivity was reduced from 1359 ± 120 to 992 ± 77 counts per 124 μm^2^ (a 27% decline, P < 0.05). Ex-4 as either a pre- or post-injury treatment attenuated the B-TBI-induced reductions of SYP immunoreactivity; pre-injury treatment numbers were 1280 ± 53 and post-injury treatment numbers were 1319 ± 67, vs. sham 1359 ± 120 (Fig. 5, Lower Left). Ex-4 treatment in the absence of blast injury had no significant effects on SYP immunoreactivity in any tissue. Representative images of cortical SYP immunoreactivity are provided in Fig. 5 (Right). Figure 5 *SYP near here*.

### Ex-4 effects in an *in vitro* model of neuronal cell injury in hippocampal derived cells

**Figure 6:**
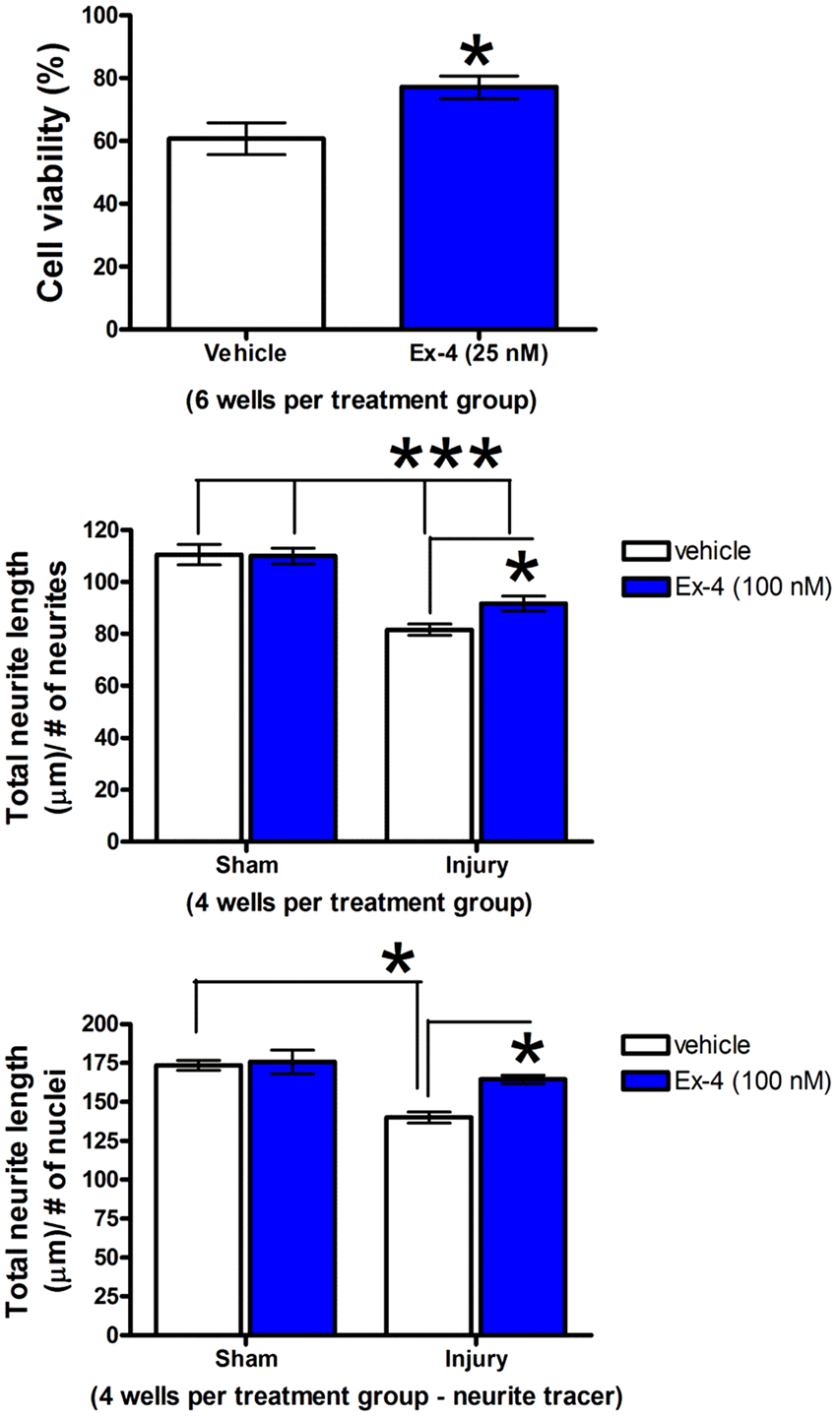
Effects of Ex-4 on HT22 cell viability and neurite length in an *in vitro* model of mild traumatic brain injury. Upper Panel: Treatment of injured HT22 cells with Ex-4 (25 nM) enhanced cell viability cells when compared to injured vehicle-treated cells (*P < 0.05). Middle Panel: Cell injury induced significant reductions in neurite number-normalized neurite length in both vehicle and Ex-4 (100 nM) treated cells (***P < 0.001). However, treatment of injured HT22 cells with Ex-4 ameliorated had less injury-induced reductions in neurite number-normalized neurite length compared to vehicle treated injured cell (*P < 0.05). Lower Panel: Cell injury induced a significant reduction in nuclei-normalized neurite length in vehicle injured cells (*P < 0.05). Treatment of injured cells with Ex-4 (100 nM) prevented the injury-induced reduction in nuclei-normalized neurite length, and led to significant preservation of neurite length compared to vehicle treated injured cells (*P < 0.05). Data are presented as a mean ± s.e.m. of measurements from 4 to 6 wells per treatment group.

The cell culturing conditions are illustrated in Fig. 1C. HT22 cells were subjected to a series of controlled pressure pulses that caused a biaxial deformation of the cells. Two hours after the final of 4 pulses, the effects of mechanical injury were evaluated on HT22 cell viability and neurite length in the presence and absence of Ex-4. In vehicle treated cells, 61 ± 5% of the cells were shown to be viable after injury. Pre-treatment with Ex-4 (25 nM) provided a significantly greater number of viable cells compared to the vehicle injury group (77 ± 4% *p < 0.05, Fig. 6 Upper Panel). Measurements of neurite length indicated that the mechanical injury induced structural changes that led to shrunken neurites (***p < 0.001, Fig. 6 Middle Panel). While treatment with Ex-4 (100 nM) did not fully abolish the changes in neurite length induced by injury, the neurites of cells treated with Ex-4 were significantly longer than those of vehicle treated injured cells (*p < 0.05, Fig. 6 Middle Panel). Figure 6 (Lower Panel) illustrates similar results for total neurite length per cell (*p < 0.05); vehicle treated injured cells had shortened neurites whereas Ex-4 treated injured cell neurites were more akin to those of the sham control group. Figure 6 *, HT22 cell data near here*.

## Discussion

Due to a greater incidence of military and civilian injury in militarized zones by improvised explosive devices, scientists and physicians are using blast models of traumatic brain injury as tools to investigate the pathological processes initiated in the brain by primary blast shockwave injuries that subsequently cause behavioral impairments in blast survivors^21, 22^. Commonly used models of blast injury involve the use of shock tube systems and open field blast systems^21–26^. Each system has advantages and disadvantages, and the choice of which model to use will depend on the focus of the each study^27^. Interestingly different neurological sequalae have been observed depending on the use of the different blast model systems. These changes in outcomes may be attributed to basic differences between open field blast and shock tube blast injury models in shockwave physical parameters, quaternary and environmental effectors following the blast^26–29^. It would be reasonable to speculate that different mechanisms of injury induction may give rise to different biological responses to similar unit forces of blast energy. In the present study, we chose to examine the effect of the FDA approved medicine exendin-4 in an open field model of blast traumatic brain injury as we believe that this form of injury is more representative of events in the battlefield^14, 16, 30^.

This study presents data illustrating that mild B-TBI-induced deficits in simple measures of cognition, and injury induced changes in synaptophysin immunoreactivity which reflects synapse density, an important marker of synaptic function and cognition^31, 32^ in mouse cortical and hippocampal tissues. The changes in recognition and working memory behaviors lasted up to 30 days after blast injury. These deficits were substantially reduced by treatment with Ex-4, when administered as either a pre- or post-B-TBI treatment strategy over 7 days. The resulting deficits in NOR and Y-maze may represent changes in mouse brain circuits caused by the secondary effects of tissue damage triggered by the blast shockwave. Studies have identified an important involvement of the hippocampus in non-spatial recognition memory function^33–37^. Similar to observations for the NOR paradigm, the Y-maze spatial memory assessment has been shown to be hippocampal-dependent^38–40^. Whereas the precise brain regions involved in mammalian object recognition have been the subject of some debate, studies have highlighted a significant role of both cortex and hippocampal brain areas for the generation of spatial and non-spatial memories (see Preston and Eichenbaum^41^, for a review on the subject). Implications of our studies are that the signaling/pathological events triggered by B-TBI are amenable to beneficial manipulation by treatment with Ex-4. In the present study, steady-state levels of Ex-4 are achieved by approximately 24 hours after implantation of the micro-osmotic pumps. This will have ensured the activation of GLP-1 receptors that mediate signaling events via a G-protein (Gα protein). This induces a rapid increase in intracellular cAMP that activates several cAMP dependent cell pro-survival pathways, such as Responsive Element Binding Protein 1 (CREB), ATF4 (Cyclic AMP-Responsive Element-Binding Protein 2 or CREB2) and AKT signaling^42–46^.

Numerous studies involving small animal models of TBI have described changes in synaptic protein levels following TBI; commonly utilized models include fluid percussion or controlled cortical injury^47–50^. These studies describe both reductions and increases in SYP protein after TBI in hippocampus and cortical regions measured over a range of times after injury, from 1 day to 30 days. Of particular relevance to our study, experiments performed *in vitro* using blast shockwaves with rat hippocampal slice cultures describe reduced SYP protein levels^51^; and deficits in long term potentiation (LTP). LTP is thought to be an electrophysiological correlate of memory, which is associated with synaptic dysfunction that can be protected by pharmacological treatments that augment cAMP signaling^52, 53^. These studies were performed from 1 to 10 days after blast injury. In our mild blast TBI model we were able to detect reductions in protein labelling in neurons of the pre-synaptic protein SYP, which are in-line with such prior studies. Indeed, Goldstein *et al*.^22^, reported that blast tube exposure induced persistent hippocampal-dependent learning and memory deficits that endured over at least 30 days and correlated with impaired axonal conduction. Our reduction of SYP protein staining was, likewise, found both in the temporal cortices and in the hippocampi of injured mice. Moreover in both regions, Ex-4 administration initiated either pre- or post-injury attenuated the B-TBI-induced reductions of the SYP protein. Additionally, as suggested by Preston and Eichenbaum^41^, likely the significantly reduced number of synapses observed in the cortical and hippocampal regions work synergistically to cause the observed cognitive deficits in NOR and Y-maze. Our observations of altered synaptophysin staining in these brain regions complement evidence of neurodegeneration seen in our earlier study with this model^16^; providing insight into cellular dysfunctions responsible for the cognitive deficits in NOR and Y-maze. Particularly notable in this earlier study were B-TBI-induced gene pathway changes associated with the development of long-term neurodegenerative disorders, such as Alzheimer’s disease, which together with other key processes were reversed by Ex-4 treatment model^16^. The lack of a group-dependent effect seen in the present EPM studies at either Ex-4 treatment time point indicates that there was no confounding animal injury or handling-induced anxiety.

Data obtained from our present *in vitro* TBI model are in-line with our prior study undertaken in the same cell line^54^. It is important to note that the beneficial actions of treatment with Ex-4 in the biaxial stretch injury model are in line with the neurotrophic and protective actions of GLP-1R agonists across a broad number of neuronal cell lines challenged with a variety of physiological and pharmacological insults^17^, and importantly may offer further evidence of a direct effect of the drug on neurons that controls for any indirect effects mediated via possible peripheral mechanisms in the animals. The dose of Ex-4 used in the present animal study was 3.5 pM/kg/min, which is approximately 21 µg/kg per day. This compares favorably to the dose used in the clinic for type II diabetes. In the clinic, Ex-4 (Exenatide long-acting release (LAR) formulation (*Bydureon*) is given at 2 mg per week subcutaneously, which translates to 3.4 µg/kg/day (85 kg human). Our study thus represents a 50% clinical dose following the normalization of body surface area between mice and humans, in accord with FDA recommendations^55^, and plasma Ex-4 levels measured in our study were not substantially different from those measured in human Exenatide studies^56^. Taken together, these studies support the repurposing of exendin-4, and other agents in this class, for ailments other than type II diabetes depending on further preclinical studies and clinical trials.

## Materials and Methods

### Animal studies, General maintenance of animals

Male mice, 30–40 g, were housed at five per home cage under a constant 12-h light/dark cycle, at room temperature (22 ± 2 °C). Animals (ICR strain) had unlimited access to food and water (Purina). For each assessment time point and treatment regimen an animal was utilized only once. The experimental protocols (M-11-086 and 331-TGB-2018) were approved by the Sackler Faculty of Medicine Ethics Committee (Tel Aviv, Israel) and the Animal Care and Use Committee of the Intramural Research Program, National Institute on Aging (Baltimore, MD, USA). All animal study methods were carried out in accordance and complied with the National Institutes of Health (DHEW publication 85–23, revised, 1995). The animal numbers for each assessment group and experimental measures were selected based upon our prior studies^14, 15, 30, 57^. All attempts were made to minimize both the numbers of mice used for our studies and any suffering.

### Induction of a mild B-TBI

Experimental conditions used to induce the mild B-TBI have been described in detail elsewhere^14, 16, 30^. Mice were anaesthetized with a combination of ketamine and xylazine (100 mg/kg and 10 mg/kg, respectively). After full anesthesia had been achieved the animals were placed on a raised platform (1 meter above ground level) in a circle. The animals were positioned 7 meters from the epicenter of a detonation^14^. The shockwave was formed by the detonation of 500 g of trinitrotoluene which was also raised 1 meter above ground level. The shockwave peak pressure was measured by pressure sensors positioned similarly to the mice. The sensors were Free-Field ICP® Blast Pressure Sensor; PCB Piezoelectronics, Depew, NY, USA, Model 137. The peak overpressure force was determined to be 2.5 PSI (17.23 kPa). Animal treatment groups were divided into: (1) sham exposed, meaning that the animals were not subjected to a blast injury (sham); (2) vehicle treated blast traumatic brain injured animals (B-TBI) and (3) drug treated blast injured animals receiving drug as either a pre-injury treatment (when treatment was initiated 48 hours before injury, Ex-4/B-TBI) or post-injury treatment (B-TBI/Ex-4) where treatment was initiated two hours after injury. Additional animals were treated with Ex-4 but with no blast injury (Ex-4). The experimental timeline for animal studies is shown in Fig. 1B.

### Drug preparation

The half-life of Ex-4 in healthy humans has been shown to be less than 2 hours after a single administration of peptide^58–60^. Due to this relatively short duration we chose to use a micro-osmotic pump drug delivery system to ensure that steady-state plasma drug levels were attained prior to and shortly after the induction of B-TBI, a critical phase of the development of neurological deficits following brain injury. The Ex-4 was delivered from a subcutaneously implanted, seven-day release Alzet micro-osmotic pump (Model 1007D, Alzet, Cupertino, CA)^15, 16, 57, 61–63^. Our use of a 7 day micro-osmotic pump to deliver sustained levels of Ex-4 hence mimicked the use of Exenatide LAR (*Bydureon*) in humans. These micro-osmotic pumps delivered drug at a constant rate from 4–6 hours from the time of implantation, and the osmotic pump delivered the drug at a rate of 3.5 pM/kg/min (21 µg/kg/day). Pumps were surgically implanted prior to, or shortly after (2 hours), the induction of B-TBI. The dose of Ex-4 was selected based upon our prior *in vivo* studies examining the effects of the drug in concussive and blast models of TBI^15, 16, 57^ and in rodent efficacy studies with a focus on neurodegeneration^61–63^. Ex-4 was prepared in equal volumes of saline and dimethyl sulfoxide (100% DMSO, SIGMA).

### Pharmacokinetic evaluation of the time dependent plasma levels of Ex-4

In a parallel set of animals micro-osmotic pumps were implanted (Model 2002 14 days, Ex-4 dose 3.5 pmol/kg/min) and plasma was derived from heparinized whole blood collected from the animals at 6, 24, 48, 80 and 168 hours after surgical implantation of the pumps. The plasma levels of Ex-4 were measured by a sensitive competitive ELISA specific for Ex-4 (Catalog #FEK-070-94, Phoenix Pharmaceuticals, Inc.), following the manufacturers protocol. In brief, the peptide standard and plasma samples were loaded onto ELISA plates; after this the primary antibody was added to each well containing standard and plasma sample. The ELISA plate was then incubated at 4 °C overnight; the next day the samples were incubated with the biotinylated Ex-4 peptide. The ELISA plate was then washed and further incubated with a streptavidin labelled HRP solution; after a series of washing steps the unknown samples were incubated with the substrate solution and read on a fluorescence plate reader using excitation/emission wavelengths of 325 nm and 420 nm. The fluorescence signals were compared to the standard curve and the concentration of plasma Ex-4 was determined (pg/ml). The experimental timeline for the pharmacokinetic assessment of plasma Ex-4 levels is shown in Fig. 1A.

### Mouse behavioral tests

Observations of behavioral deficits in animals subjected to this mild B-TBI model were described by Rubovitch and co-workers^14^, changes in behavior were reported to be present starting from day 7 and persisted to day 30 after injury. Due to these initial observations, we chose to assess animal behaviors from day 7 and 30 post-injury. A battery of behavioral challenges was initiated on day 7 and 30 after the induction of B-TBI. Mouse cognitive tests were performed using an unbiased operator basis. The assessments employed were as follows: YM, NOR and the EPM. Our laboratories utilize these behavioral paradigms routinely^57, 64–66^. All behavioral studies were performed during the light phase of the light/dark cycle. The animal numbers utilized in the behavioral studies are provided in Tables 1 and 2.

**Table 1:** Pre-injury animal treatment groups.

Behavioral test	Testing initiation day 7 after B-TBI			
	Sham	B-TBI	Ex-4/B-TBI	Ex-4
Y maze	n = 19	n = 25	n = 16	n = 11
Novel Object Recognition	n = 9	n = 10	n = 11	n = 9
Elevated Plus Maze	n = 16	n = 17	n = 16	n = 11
**Behavioral test**	**Testing initiation day 30 after B-TBI**			
	**Sham**	**B-TBI**	**Ex-4/B-TBI**	**Ex-4**
Y maze	n = 14	n = 14	n = 16	n = 14
Novel Object Recognition	n = 9	n = 10	n = 10	n = 10
Elevated Plus Maze	n = 13	n = 14	n = 12	n = 12

The animal numbers were as follows when Ex-4 was administered prior to the blast injury.

**Table 2:** Post-injury animal treatment groups.

Behavioral test	Testing initiation day 7 after B-TBI			
	Sham	B-TBI	B-TBI/Ex-4	Ex-4
Y maze	n = 20	n = 20	n = 16	n = 19
Novel Object Recognition	n = 11	n = 13	n = 13	n = 11
Elevated Plus Maze	n = 16	n = 13	n = 16	n = 19
**Behavioral test**	**Testing initiation day 30 after B-TBI**			
	**Sham**	**B-TBI**	**B-TBI/Ex-4**	**Ex-4**
Y maze	n = 14	n = 16	n = 16	n = 14
Novel Object Recognition	n = 8	n = 13	n = 14	n = 15
Elevated Plus Maze	n = 13	n = 16	n = 17	n = 6

The animal numbers were as follows when Ex-4 was administered after the blast injury.

### Y Maze Protocol

The YM task is an assessment commonly used to evaluate spontaneous exploration and responsiveness to novel environments and spatial working memory function. The test apparatus is constructed out of identical black Plexiglass arms (8 × 30 × 15 cm) where the arms extend from a central point at a 120° angle from the center. Inside each arm is a different spatial cue designed to give the mouse a visual memory anchor. Typically, there are two trials undertaken a few minutes apart; here it was at 5 minute intervals. For each first trial the start arm is selected randomly. Each animal is placed into the center point of the YM environment; during the first 5 minute trial, one of the two arms is randomly closed, during the second 2 minute trial all three arms are open for exploration. The total amount of time the mouse explored in each arm during the second trial is recorded. To avoid any possible confounds, between trials the maze is thoroughly cleaned. The time spent in the novel previously unexplored arm over the familiar previously explored arm is used to assess for any behavioral differences between each animal treatment group i.e. (the time spent in new arm minus time spent in familiar arm)/(time spent in new arm plus time spent in familiar arm).

### Novel Object Recognition Protocol

The NOR task is routinely used to evaluate recognition memory in rodents. Non-compromised rodents display an inherent tendency to explore novel objects in their immediate locations. This feature of mouse behavior allows for the assessment of visual recognition memory function. Perhaps more importantly it also allows the assessment of the effects of different stimuli on this inherent activity. The test utilizes two trials where for the first trial animals are allowed to examine two objects for a defined amount of time, herein 5 minutes. The second trial takes place 24 hours after the first, in which the animals are challenged with two objects where one is the same as in the first trial and the other is new to the animal. In the second trial mice are also allowed to explore the objects for 5 minutes. A discrimination preference index is calculated and used to evaluate the animal’s recognition memory. The index is calculated by the following: the time the animal spends near the novel object minus the time spent near the familiar object, divided by the sum of the time near the novel and familiar objects. These methods have been described elsewhere^64, 65, 67–70^.

### Elevated Plus Maze Protocol

The EPM test provides a measure of fear in rodents. This assessment relies on the natural anxiety-like emotions exhibited by rodents when challenged with brightly lit environments. The apparatus is in the shape of a plus formation (+), with arms extending from the center at 90° angles from each other. Opposite arms in the plus formation are identical i.e. for the low open ‘unsafe’ arms the walls are 30 cm long × 5 cm wide × 1 cm high and the other two ‘safe’ arms have high walls 30 cm long × 5 cm wide × 15 cm high; all arms have open tops. To induce additional stress to the animals the maze is raised above floor level by 50 cm. The high ‘safe’ and low ‘unsafe’ arms are made of black and white acrylic glass, respectively. This assessment utilizes a single trial where animals are placed in the middle the EPM facing one of the open ‘safe’ arms. The trial lasts for 5 minutes where the total time the mouse spends in all of the arms is recorded and used statistically to identify any treatment-dependent changes in fear-like behavior.

### Assessment of hippocampal and temporal cortex synaptophysin immunoreactivity levels day 3 after injury

Brain tissues in B-TBI animals manifested evidence of neurodegeneration 3 days after injury in a separate study^16^. As a consequence of these observations we chose to study potential B-TBI-induced alterations in synaptophysin immunoreactivity in hippocampal and cortical tissues on day 3 after injury. Immunohistochemistry studies were performed on mouse hippocampal (HIPP) and temporal cortex (CTX) tissue sections obtained from animals euthanized 3 days after the blast injury. B-TBI-induced changes in synaptophysin (SYP) immunoreactivity levels were used as an index of neuronal synapse numbers. Staining methods used were similar to that described by Masliah and co-workers^71^. The procedures used to prepare mouse brain tissues have been described in detail elsewhere^30, 57^. Animals were deeply anaesthetized using a combination of ketamine (100 mg/kg) and xylazine (10 mg/kg). They were then perfused transcardially with 10 ml of phosphate buffered saline (PBS) followed by perfusion with 20 ml of a paraformaldehyde (PFA 4%) in buffer. Intact mouse brains were further incubated overnight in a 4% PFA in buffer and then transferred to 1% PFA buffer. Finally, the brains were submerged in 30% sucrose for two days prior to sectioning. Sections (30 µm) were prepared and every 12^th^ section was stained with a primary antibody that detects SYP (Santa Cruz, sc-9116). After the incubation with primary antibody the sections were rinsed and exposed to a fluorescent dye labeled secondary antibody (Jackson ImmunoResearch, Cat #111-585-008, at a dilution of 1:300). The probed brain sections were affixed to 2% gelatin coated glass slides and SYP immunoreactivity was assessed in a blinded manner to avoid any possible operator bias. Analysis was performed by Imaris software (Bitplane AG, Zurich, Switzerland) using conditions similar to those described by Schätzle and co-workers^72^, detection thresholds were set to count round, synapse structures. The slides were observed using a Zeiss LSM 510 confocal microscope (Carl Zeiss, Jena, Germany). The SYP immunoreactivity from different areas was used to generate an index of the number of synapses for each tissue region. The numbers of SYP positive staining synapses from a series of sections, 4 to 5 sections per animal, were averaged to provide a mean measurement per animal. The average measurements from each animal were used to generate an overall treatment group mean that was used for statistical analysis. Animal treatment groups were as follows, for the temporal cortical region: sham n = 5; B-TBI n = 4; B-TBI/Ex-4 n = 4; Ex-4/B-TBI n = 6; Ex-4 n = 4. For the hippocampal region: sham n = 6; B-TBI n = 8; B-TBI/Ex-4 n = 4; Ex-4/B-TBI n = 7; Ex-4 n = 4.

### Statistical analysis–mouse behavioral tests and assessment of synaptophysin immunoreactivity

Measurements are presented as mean ± S.E.M.. Statistical differences were assessed with SPSS 17 software (Genius Systems, Petah Tikva, Israel). Behavioral measures were assessed with ANOVA (one way); p values of *post hoc* comparisons were evaluated using the Fisher LSD or Tukey’s HSD tests. A minimal statistical significance level of 0.05 was required.

### Cell culture studies, *In vitro* model of neuronal cell injury in hippocampal derived cells

The hippocampal derived cell line HT2219,20 was utilized in an *in vitro*, sub-lethal stretch TBI model of cellular injury using conditions similar to those described by others^73–75^. The cells were maintained in Dulbecco’s Modified Eagle Medium (DMEM) containing 10% fetal bovine serum (Gibco, Carlsbad, CA) and 1% penicillin/streptomycin (Amresco, Solon, OH). Cells were grown in a humidified incubator with 5% CO_2_ at 37 °C. A biaxial stretch injury was performed on HT22 cells. Essentially this involved the formation of a physical deformation of the cell in two different axes. HT22 cells were seeded (15,000 cells per well) in 24 well HT BioFlex plates coated with Collagen Type I (Flexcell International Corp., Burlington, NC). Cell injury was induced with a Cell Injury Controller II (Virginia Commonwealth University, Richmond, VA)^54, 76, 77^. The Cell Injury Controller II applies a short duration nitrogen pulse (99 ms) to each well to distort the cells attached to the membrane. The pulse injury pressure (PI) generated by the instrument can be regulated over a range of intensities; in the present study, the PI was approximately 7 psi.

Plated cells were equilibrated overnight, and the following day the cells were exposed to vehicle or Ex-4 (25 or 100 nM). After a 2 hour incubation the cells were challenged with the first of four PIs; each pules was separated by a 1 hour interval. Two hours after the last PI, measurements were made on cell viability and injury-induced changes in neurite length. Based upon prior studies and a comparison with the literature our present experimental parameters correspond to a mild injury^54^. The experimental timeline for HT22 cell studies is shown in Fig. 1C.

### HT22 cell viability

Cell viability was determined using the ReadyProbes Cell Viability Imaging Kit (Molecular Probes, Eugene, OR). The assessment of the numbers of living and dead cells is based upon the incorporation of two fluorescent markers, one that indicates living cells (NucBlue® Live) and the other dead cells (NucGreen® Dead). Two hours post-injury the stains were added to the media in each well. The cells were imaged following a short incubation at room temperature using an Olympus FluoView 1000 confocal microscope. Six replicates were used for each treatment group and one image from the center of each well was analyzed. Live and dead cells were counted automatically using the Analyze Particles routine in ImageJ with identical parameters.

### HT22 cell neurite length

Two hours post-injury, the cells were subjected to a series of staining procedures used to measure neurite lengths. Injury-induced changes in neurite length were assessed by use of the Neurite Outgrowth Staining Kit (ThermoFisher Scientific) in addition to Hoechst nuclear staining. Cell membranes were visualized by exposing the cells to a stain combined with 3.7% formaldehyde in phosphate buffered saline. The cells were incubated with the mixture at 37 °C. After a short incubation period the solution was discarded and replaced with a background suppression dye containing Hoechst (2.5 µg/ml). Images were captured using an Olympus FluoView 1000 confocal microscope. For each experiment, there was a minimum of two images captured from 4 wells for each treatment group. Neurites were counted using the ImageJ program with the aid of the NeuronJ Plugin to provide semi-automated tracing. In an additional parallel set of experiments the Neurite Tracer Plugin was utilized to provide fully automated neurite measurements, as has been used previously^78^. Nuclei counts were performed manually in ImageJ for normalization with Neurite Tracer measurements to determine average total neurite length per cell.
